# Sero-epidemiological survey of bluetongue disease in one-humped camel (*Camelus dromedarius*) in Kassala State, Eastern Sudan

**DOI:** 10.1186/s13620-021-00186-2

**Published:** 2021-03-26

**Authors:** Molhima M. Elmahi, Mohammed O. Hussien, Abdel Rahim E. Karrar, Amira M. Elhassan, Abdel Rahim M. El Hussein

**Affiliations:** 1 Kassala Veterinary Research Laboratory, Animal Resources Research Corporation (ARRC), P.O. Box 237, Kassala, Sudan; 2 Central Laboratory, Ministry of Higher Education and Scientific Research, P.O. Box 2081, Khartoum, Sudan; 3 Faculty of Veterinary Medicine, University of Khartoum, Ministry of Higher Education and Scientific Research, P.O. Box 32, Khartoum North, Sudan; 4 Central Veterinary Research Laboratory (CVRL), Animal Resources Research Corporation (ARRC), P.O. Box 8067, (El Amarat), Khartoum, Sudan

**Keywords:** Epidemiology, Survey, Camels, BTV, cELISA, Sudan

## Abstract

**Background:**

Bluetongue (BT) is a vector-borne viral disease of ruminant and camelid species which is transmitted by *Culicoides* spp. The causative agent of BT is bluetongue virus (BTV) that belongs to genus *Orbivirus* of the family *Reoviridae*. The clinical disease is seen mainly in sheep but mostly sub-clinical infections of BT are seen in cattle, goats and camelids. The clinical reaction of camels to infection is usually not apparent. The disease is notifiable to the World Organization for Animal Health (OIE), causing great economic losses due to decreased trade and high mortality and morbidity rates associated with bluetongue outbreaks. The objective of this study was to investigate the seroprevalence of BTV in camels in Kassala State, Eastern Sudan and to identify the potential risk factors associated with the infection. A cross sectional study using a structured questionnaire survey was conducted during 2015–2016. A total of 210 serum samples were collected randomly from camels from 8 localities of Kassala State. The serum samples were screened for the presence of BTV specific immunoglobulin (IgG) antibodies using a competitive enzyme-linked immunosorbent assay (cELISA).

**Results:**

Seropositivity to BTV IgG was detected in 165 of 210 camels’ sera accounting for a prevalence of 78.6%. Potential risk factors to BTV infection were associated with sex (OR = 0.061, *p*-value = 0.001) and seasonal river as water source for drinking (OR = 32.257, *p*-value *=* 0.0108).

**Conclusions:**

Sex and seasonal river as water source for drinking were considered as potential risk factors for seropositivity to BTV in camels. The high prevalence of BTV in camels in Kassala State, Eastern Sudan, necessitates further epidemiological studies of BTV infection in camels and other ruminant species to better be able to control BT disease in this region.

## Background

Bluetongue (BT) is an infectious disease of ruminants caused by bluetongue virus (BTV) which belongs to the Orbivirus genus of the family Reoviridae with at least 28 recognized serotypes [1]. In sheep and certain species of deer, the clinical disease is most common [1] whereas cattle, goats and camelids usually have sub-clinical disease [2–4]. Bluetongue virus transmission from animal to animal requires insect vectors. *Culicoides* midges are the main vectors of the virus, with *C. imicola* being the main vector species in Africa and southern Europe [1]. At least, four BTV serotypes designated as serotypes 1, 2, 4 and 16 are enzootic in different states of Sudan [5, 6]. These serotypes (1,4 and 16) were recovered from sentinel calf herds at Shambat (Khartoum) [5]. The sheep breeds, such as Sudanese ecotypes of sheep, may develop clinical disease while cattle and camelids mostly develop sub-clinical infections [1]. Similarly, the clinical reaction of camels to disease agents such as Foot and mouth disease (FMD), Rinderpest, Bovine viral diarrhea (BVD, Rift valley fever (RVF), Parainfluenza-3 (PI3) and Bovine herpes virus-1 (IBR) is usually not apparent nor is it expected, and illness may pass unnoticed [7]. In Sudan, Abu Elzein [8] reported that susceptibility of camels to BTV infection was lower than that of other ruminant species. Generally, BTV infection is widely prevalent in camels in Sudan as indicated by Abu Elzein [8] who reported an overall seroprevalence of 16.6%. Saeed [9] reported that BTV antibodies in camel were highly prevalent (66.8%) in Khartoum State, Sudan. Also Elhassan et al. [10] reported that BTV antibodies in cattle were highly prevalent in Gazira (central Sudan), and cattle can be source of infection for *Culicoides* species that are biologically transmitted BTV to other ruminants. Furthermore, Adam et al. [11] and Khair et al. [12] reported that BTV existed in North Kordufan and East Darfur States in western Sudan, respectively.

Kassala State is located in the eastern part of Sudan. It has borders with Eritrea and to other Sudanese states (Red Sea State, River Nile State, Khartoum State and Gedarif State). The animals naturally move among Sudan and Eritrea, therefore during this movement, the animals share common water and pasture routes. In Kassala State, research on camel diseases other than Trypanosomiasis and brucellosis is very limited, despite the fact that camels play an increasingly important role in the livestock economy of the Sudan. At present, little is known about the prevalence and associated risk factors of BTV infection in camels in Sudan in general and in Kassala State in particular. The present study aimed to provide the first serological investigation of BTV infection and to identify potential risk factors associated with the infection among one-humped camels (*Camelus dromedarius*) in Kassala State, Eastern Sudan.

## Methods

### Study area

**Fig. 1:**
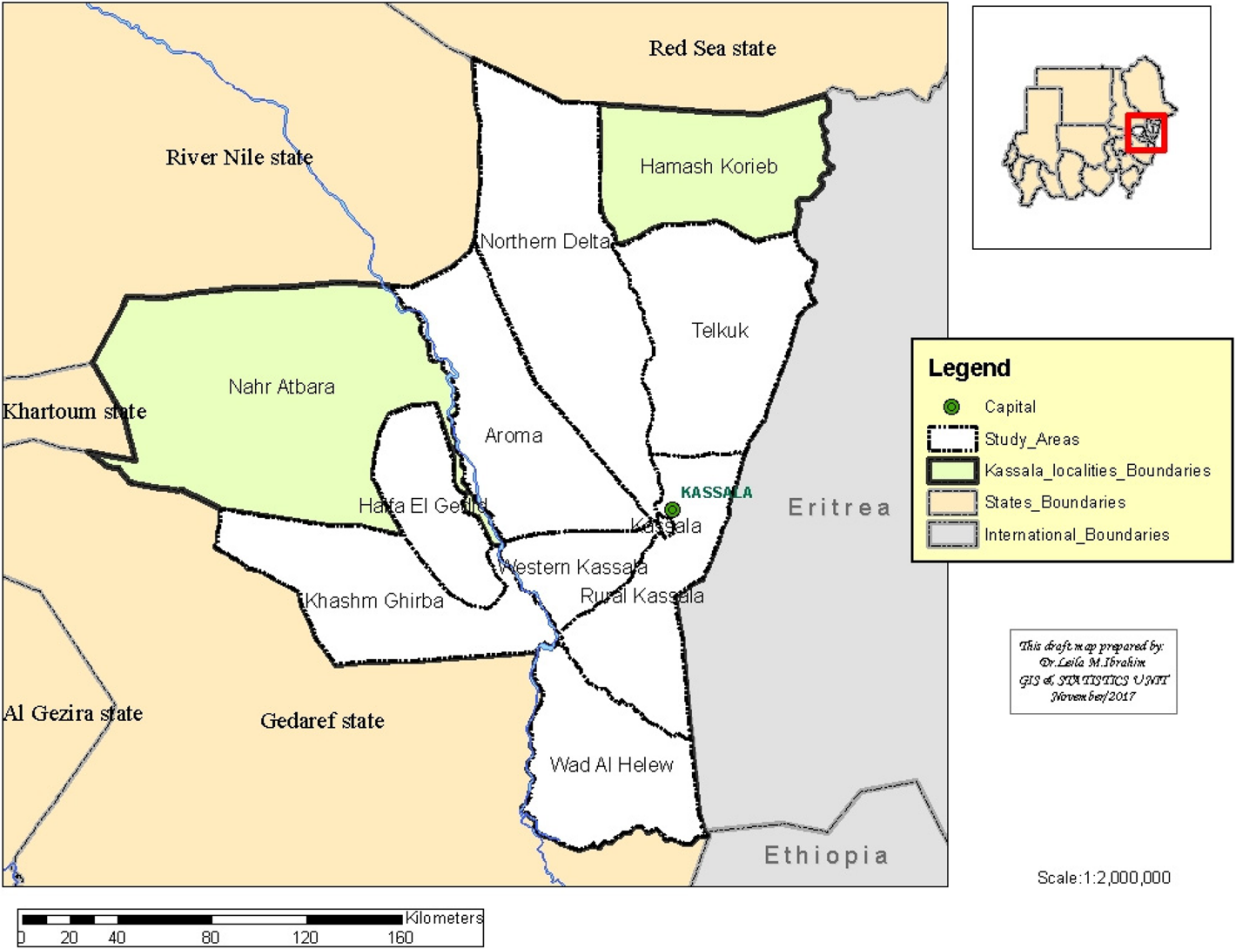
Map of Kassala State, Sudan showing the locations where serum samples were collected

Kassala State covers an area of 42,282 km^2^, which located between latitudes 14°N and 17°N and longitudes 34°E and 37°E. It is divided into 11 localities as depicted in Fig. 1. The State features poor savanna in the north and east and rich savanna in south and west.

### Study design

A cross sectional study was conducted to estimate the prevalence of BTV-specific IgG antibodies in camels and to study risk factors associated with the BTV infection. A multistage probability sampling method was used in this study. Sample size for the study was estimated using the formula: *n* = Z^2^PQ/L2 [13], where n is the required number of individuals to be examined; Z is a constant = 1.96; P is known or estimated prevalence; Q = (1 − P); and L is the allowable error. A total of 216 animals were estimated using this formula assuming 16.6% prevalence reported by Abu Elzein [8]. A total of 210 samples were randomly collected from camels in eight localities including two localities from the north section (Aroma and Northern Delta), three from the south (Rural Kassala, Wad Al Helew and Khashm Ghirba), one locality from the north east (Telkuk) and two from the west section (Western Kassala and Halfa) during 2015–2016 (Fig. 1). The sample size tested was slightly lower than calculated because many camel owners refused to donate animal’s blood. Villages were selected from each of the 8 localities mentioned above, based on a strategy that sought to cover all localities. Finally, simple random sampling was applied to choose the animals from each village. All camels included in this study were aged 1 year and > 1 year-old. Animals sampled were of both sexes and from local breeds including Bushari and Anafi. They were sampled at different seasons including winter (December–February), summer (March–May) and rainy season (July–October) (Table 1).

**Table 1:** The univariable association between potential risk factors and BTV seropositivity among camels in Kassala State, Eastern Sudan using the chi-square test

Factor	Variable	Animals tested	Animals affected (%)	***p-***value
**Section**	West	98	67 (68.3%)	
	South	55	49 (89.0%)	0.004^*^
	North	45	37 (82.2%)	
	North East	12	12 (100%)	
**Locality**	Halfa	20	16 (80%)	
	Western Kassala	78	51 (65.3%)	
	Khashm Ghirba	9	7 (77.7%)	
	Wad Al Helew	25	25 (100%)	0.003^*^
	Aroma	12	12 (100%)	
	Northern Delta	33	25 (75.7%)	
	Telkuk	12	12 (100%)	
	Rural Kassala	21	17 (80.9%)	
**Sex**	Female	82	80 (97.5%)	0.000^*^
	Male	128	85 (66.4%)	
**Age (year)**	1	23	17 (73.9%)	0.366
	> 1	187	148 (79.1%)	
**Breed**	Bushari	208	163 (78.3%)	0.617
	Anafi	2	2 (100%)	
**Season**	Rainy	109	77 (70.6%)	
	Summer	38	37 (97.3%)	0.002^*^
	Winter	63	51 (80.9%)	
**Herd Size**	Small	45	40 (88.8%)	0.057
	Medium	165	125 (75.7%)	
**Ecology**	Rich Savana	133	100 (75.1%)	0.080
	Savana	77	65 (84.4%)	
**Water source**	Canal	20	16 (80%)	
	Hafeer	78	51 (65.3%)	
	Atbara river	35	33 (94.2%)	0.000*
	Water station	6	2 (33.3%)	
	Seasonal river	28	27 (96.4%)	
	wells	43	36 (83.7%)	

*Significantly different, with a *p*-value ≤0.05

### Questionnaire

Owners of flocks were interviewed using a structured questionnaire. Information was collected for bluetongue disease. In addition, a range of other data were collected about each animal including sex, age, breed (Anafi and Bushari), movement, biting flies vector (presence or absence), water source, co-rearing with other animal species, and herd size.

### Collection of blood samples

Blood samples were collected in plain vacutainer tubes, left overnight to clot at + 4 °C. The serum was then collected by centrifugation at 2000 rpm for 15 min and kept at − 20 °C until being tested for BTV antibodies by cELISA.

### Competitive enzyme – linked Immunosorbent assay (cELISA)

A total number of 210 serum samples were tested using competitive ELISA Kits (IDEXX, USA). The procedure was conducted according to the manufacturer’s instructions. The tested sera were considered positive when they produced an optical density less than or equal to 70% of the mean of the negative controls (S/N). The tested sera that produced an optical density greater than or equal to 80% of the mean of the negative controls (S/N) were considered negative and the tested sera that produced an optical density greater than 70% and less than 80% of the mean of the negative controls were considered doubtful and were retested.

### Statistical analyses

The serological results and other information gathered during this investigation were compiled and managed using descriptive analysis. The statistical computation was performed using statistical package SPSS version 20 (SPSS Inc., Chicago, U.S.A.). To identify the associations of the risk factors with BTV seroprevalence, the chi square (X^2^) test was used in a univariable analysis. A multivariable model for the outcome variable was established using logistic regression analysis. Odd ratios and 95% confidence interval (CI) were determined and risk factors with a *p-*value ≤0.05 were considered to have a significant association to BTV seropositivity.

## Results

Out of 210 camel tested, 165 (78.6%) animals were found positive for BTV antibodies. None of the animals had clinical signs suggestive of BTV infection. Regarding different localities, the highest prevalence of BTV was recorded in Wad elhelow, Aroma and Telkuk (100%), while the lowest prevalence was recorded in the Western Kassala locality (65.3%) (Table 1). The prevalence was 97.5% (80/82) in females and 66.4% (85/128) in males. Animals aged above 1 year showed a prevalence of BTV infection of 79.1% (148/187) compared to those aged less than 1 year (73.9%). According to breed, the prevalence was highest 100% (2/2) in Anafi breed. The infection with BTV was highest in the summer season (97.3%) while it was lowest in rainy season (70.6%) and intermediate during winter season (80.9%) (Table 1).

The results of univariate analysis using chi-square test were shown in (Table 1). The final model of BTV infection revealed that only two independent risk factors were statistically significant. Male camels were less likely to be infected with BTV (OR = 0.061, CI = 0.012–0.309, *p*-value = 0.001) compared to females. Seasonal river as a water source for drinking increased the risk of contracting BTV compared to other sources of water (OR = 32.257, CI = 1.821–571.445, *p*-value *=* 0.0108) (Table 2).

**Table 2:** Multivariate analysis using logistic regression model for significant association (*p* < 0.05) of risk factors and BTV seropositivity among camels in Kassala State, Eastern Sudan

Risk factor	OR	95% CI Lower- upper	***P***-Value
**Locality**			
Khashm Ghirba	Reference	–	–
Halha	1.143	0.168–7.762	0.891
Western Kassala	0.540	0.105–2.780	0.461
Wad Al Helew	6.857	0.539–87.279	0.138
Rural Kassala	1.214	0.179–8.217	0.842
Northern Delta	0.893	0.153–5.198	0.900
Telkuk	3.143	0.238–41.507	0.384
Aroma	3.143	0.238–41.507	0.384
**Sex**			
Female	Reference	–	–
Male	0.061	0.012–0.309	0.001*
**Season**			
Summer	Reference	–	–
Rainy	0.113	0.009–1.456	0.095
Winter	0.139	0.014–1.405	0.094
**Water Source**			
Water station	Reference	–	–
Canal	3.697	0.166–82.100	0.408
Hafeer	6.470	0.424–98.653	0.179
Atbara river	7.090	0.330–152.498	0.211
Seasonal river	32.257	1.821–571.445	0.018*
Wells	7.029	0.831–59.483	0.074

*p-value ≤0.05 is significantly different

## Discussion

We conducted this study to increase knowledge about bluetongue disease in camels in Kassala State, Eastern Sudan and to study the role of camels in the epidemiology of the disease. The study revealed that IgG antibodies against BTV were highly prevalent in camels in Kassala State. In this study, the overall seroprevalence of BTV was 78.6%. As there is no vaccination program of BTV in Sudan, this high seroprevalence of BTV infection in Kassala State can be hypothesized to reflect natural infection of the camels evaluated.

Several studies in Sudan and other countries have reported seropositivity of BTV in dromedaries. Briefly, the overall seroprevalence of BTV in camels in the present study (78.6%) was higher than the previous studies in Sudan. Saeed in Khartoum State [9] reported a prevalence of 66.8%. Abu Elzien [8] reported a prevalence that varied from 0 to 40.2% (average 16.6%) in different localities in Sudan, with a 4.3% prevalence in Kassala locality. It is however, lower than that reported in camels in Gadarif State, Eastern Sudan (96.7%) [Hamad Personal communication]. In the present study, the prevalence of BTV group specific antibodies in camels in Kassala State was much higher than the 25.7% prevalence reported by Yousef et al. [14] in Saudi Arabia and the 67.8% recorded by Mozaffari et al. [15] in Iran. The high prevalence of BTV in camels in Kassala State, Eastern Sudan indicates the wide prevalence of vector midges which transmit the infection. In addition, Kassala State shares long international borders with Eritrea with no strict restriction on animals’ movement across the borders which may allow introduction of infected animals into neighboring localities.

In the present study, the prevalence varied from one locality to another. The highest prevalence of infection was recorded in Aroma, Telkuk and Wad Al Helew (100%), which may be attributed to rearing of other animals such as cattle, sheep and goats (with BTV seroprevalence of 94.6, 84.5 and 100%, respectively) with herds of camels [16], and the favorable climatic conditions conducive to survival of vector in these localities. In comparison, the Western Kassala locality recorded the lowest rate of infection (65.3%) which may be due to the separation of camel’s population from other animals and the large camel population in this area.

When assessing sex as a risk factor, there was a significant association between BTV seropositivity and sex, with seroprevalence higher in females than males. This is in agreement with Mahmoud et al. [17] in Libya. In contrast, the result of this study disagrees with Hamad [Personal communication] who found an equal seroprevalence of BTV in both sexes without significant difference. This variation may be due to the difference in the sample size of male and female or due to differences in husbandry practices.

The present study showed that water source for drinking is another potential risk factor that affects BTV seropositivity in Kassala State, with camels that drink from the seasonal river more significantly infected than camels that drink from other water sources. This may indicate that seasonal river water sources are favorable for the breeding and survival of *Culicoides* spp. vectors which may increase BTV transmission and consequently the prevalence of infection [18].

On the other hand, the risk assessment indicates that there was no significant association between seroprevalence and age of animal and ecology of the area. This is in line with the finding reported by Hamad [Personal communication] in camels in neighboring Gadarif State.

The results also showed that BTV infection rate was highest in small herd size of camels reared with other animals (88.8%), while the medium herd size of camels that are reared alone showed the lowest rate of infection (75.71%). This is in agreement with Saeed (2017) [9] who reported significant association (*p = 0.01*) between the presence of other animals and BTV seropositivity in camels in Khartoum State, Sudan. This may imply that camels acquire infection more easily when reared with more susceptible species like cattle, sheep and goats which herein showed BTV seroprevalence of 94.6, 84.5 and 100%, respectively [16].

Indeed, it is now well recognized that cattle play a crucial role in BTV epidemiology. The extended viremia and attractiveness to the vector (*Culicoeides* spp) make cattle the major reservoir and carrier of the virus [19, 20]. Moreover, significant correlation between the density of cattle and the rate of BTV infection in other animal species was recently reported in Iran [21]. Consistent with this hypothesis, cattle population in Kassala State is 786.261 which is higher than camel population (310.570) [22]. Furthermore, the high prevalence (94.6% (243/257) of BTV in cattle reared with camel was determined in the same study area [16]. Thus, infected *Culicoides* spp. that had previously been infected by feeding on BTV infected cattle could transmit the virus while feeding on the co-reared camels. The limitation of this study is that the sample size was relatively low this to the objection of camel owners to bleed their animals.

## Conclusions

In conclusion, BTV antibodies are highly prevalent in camels in Kassala State. To decrease the spread of infection, camels should be raised far from other animals whenever possible. The specific BTV serotypes circulating in the region and the persistence of BTV in camels remain to be studied. In addition, entomological surveillance of biting *Culicoides* midges involved in the transmission of BTV, and studies of their ecology and epidemiology in the area should also be carried out to better understand the epidemiology of bluetongue infection in Kassala State. This can assist policy-makers when formulating strategies to control outbreaks of bluetongue disease in this area.

## Data Availability

Data and materials are available upon request by the corresponding author.

## Abbreviations

BTV: Bluetongue virus
cELISA: Competitive enzyme-linked immunosorbent assay
IgG: Immunoglobulin G
OIE: World Organisation for Animal Health
